# A model to understand HIV-related stigma and the psychosocial well-being of children orphaned by AIDS: a theory generative approach

**DOI:** 10.1080/17290376.2021.1989023

**Published:** 2021-10-15

**Authors:** Z. Yassin, C. Erasmus, J. Frantz

**Affiliations:** a Child and Family Studies, Department of Social Work, University of the Western Cape, Cape Town, South Africa; b Department of Research and Innovation, University of the Western Cape, Cape Town, South Africa

**Keywords:** HIV-related stigma, children orphaned by AIDS, psychosocial well-being, model development, theory generative approach

## Abstract

HIV-related stigma has negatively impacted the psychosocial well-being of children who have been orphaned by AIDS-related causes. Response to reducing stigma and ensuring child well-being is hindered by the limited understanding of HIV-related stigma and how it affects the psychosocial well-being of children. Due to the call for a comprehensive understanding of HIV-related stigma, this study aimed to develop a model to understand the manner in which HIV-related stigma affects the psychosocial well-being of children orphaned by AIDS. The study implemented a mixed method, exploratory, sequential design within a theory generative approach that included concept development, statement development, model description, and model evaluation. The developed model indicated that HIV-related stigma is embedded in social interaction and mediated by children orphaned by AIDS response to stigma. HIV-related stigma and maladaptive coping strategies collectively affect several domains of child psychosocial well-being and elevate psychosocial distress. This is the first model to provide a child-centred understanding of HIV-related stigma and its consequences for psychosocial well-being. The model may be used to guide future research and inform the development of appropriate interventions.

## Introduction

Stigma has been recognised as a key driver of the human immunodeficiency virus and the acquired immunodeficiency syndrome (HIV/AIDS) pandemic (Treves-Kagan et al., 2015). HIV/AIDS’s association to premature mortality, debilitation, and behaviour deemed immoral and deviant within society has resulted in a second epidemic of HIV-related stigma that is both malicious and long-term (Link & Phelan, 2001; Parker & Aggleton, 2003; Treves-Kagan et al., 2015). HIV-related stigma has been defined as a social phenomenon by which an individual is considered to possess a discrediting attribute and thus deemed tainted, spoiled, or flawed by others (Stutterheim et al., 2009, p. 2354). The definition of HIV-related stigma offered by Stutterheim et al. (2009) draws on Goffman’s (1963) conceptualisation of stigma. Goffman (1963) described three forms of stigma: (1) abnormalities of the body, such as deformations or physical signs of illness; (2) stigma associated with blemishes of the individual character, referring to individuals engaging in immoral activities or exhibiting immoral behaviour such as promiscuity or dishonesty; and (3) tribal stigma, which signifies the stigmatisation of an individual as a result of their membership to a social group or individual.

People living with HIV/AIDS (PLWHA) are likely to be subjected to experiences of HIV-related stigma, including prejudicial actions received from family members, colleagues, employers, health care providers, communities, and governments (Dahlui et al., 2015). While focus is maintained on the adult population, current evidence has suggested that HIV-related stigma stretches beyond infected individuals to exhibit a negative outcome for their families and children. This is supported by the increase in evidence focusing on the impact of HIV-related stigma on the well-being of children who have been orphaned by AIDS (COA) (Campbell, Skovdal, Mupambireyi, & Gregson, 2010; Cluver et al., 2013; Sherr et al., 2014; Skovdal, 2012).

The stigmatisation of COA by extended family, peers, communities, and health and social services has been documented consistently (Chi, Li, Zhao, & Zhao, 2014; Deacon & Stephney, 2007; McAteer et al., 2016; Mo, Lau, Yu, & Gu, 2015; Zhao, Li, Zhao, Zhang, & Stanton, 2012). HIV-related stigma functions to undermine the well-being of COA leading to reduced access to support, increased poverty and exploitation, victimisation and bullying, poor mental health, and increased psychosocial distress (Campbell et al., 2010). Poor psychological health outcomes were similarly related to experiences of HIV-related stigma such as depression, anxiety, adjustment issues and psychological distress (Chi et al., 2014; Cluver, Gardner, & Operario, 2008; Mo et al., 2015; Zhao et al., 2012). Additionally, HIV-related stigma has been identified to impede the social well-being of COA. HIV-related stigma serves to inhibit perceived social support and leads to the impairment of healthy social attachments and the formation of healthy trusting relationships with others (Chi et al., 2014; Sherr et al., 2014).

While there has been a global call for an adequate response to reduce HIV-related stigma, the phenomenon remains poorly understood (Stangl, Lloyd, Brady, Holland, & Baral, 2013). HIV-related stigma has long been regarded as culturally and contextually specific and too sensitive to be addressed in a meaningful way. Ogden and Nyblade (2005) suggest that HIV-related stigma shares common traits across diverse contexts and cultures presenting viable avenues for addressing HIV-related stigma through the development of appropriate interventions and programmes. Varying understandings of HIV-related stigma and the lack of an appropriate framework outlining the manner in which individuals are affected by their experiences of HIV-related stigma forms a significant barrier to understanding and adequately addressing HIV-related stigma to produce better outcomes for orphaned children (Campbell & Gibbs, 2016; Genberg et al., 2009). This study, therefore, aimed to develop a model to understand the manner in which HIV-related stigma affects the psychosocial well-being of COA.

## Process of model development

A mixed method, exploratory, sequential design grounded in a theory generative approach was implemented for model development as undertaken in this study. The design followed the four stages of theory generation as proposed by Chinn and Kramer (2008), Walker and Avant (2005), and Dickoff, James, and Wiedenbach (1968), which includes: (1) concept development, (2) statement development, (3) model description, and (4) model evaluation.

### Concept development

Concepts are the basic building blocks that form the fabric of a theory. A ‘concept’ is defined as a ‘mental image of a phenomenon, an idea, or a construct in the mind about a thing or action’ (Walker & Avant, 2005, p. 59). This study adopted a concept synthesis strategy as suggested by Walker and Avant (2005) following the results of a systematic review and qualitative exploratory study for the (1) identification, (2) classification, and (3) defining of concepts.

#### Concept identification

Concepts were extracted from the first two empirical phases of the study, namely, (1) a systematic review (Yassin, Erasmus, & Frantz, 2018), and (2) a qualitative, exploratory, descriptive study (Yassin, Erasmus, & Frantz, 2019). The systematic review acted as a literary synthesis towards concept development and systematically reviewed existing literature focusing on the relationship between HIV-related stigma and the psychosocial well-being of COA. A systematic review methodology following the Preferred Reporting Items for Systematic Reviews and Meta-analysis (PRISMA) design was implemented to identify published studies which explored the concepts of HIV-related stigma and psychosocial well-being of COA (Yassin et al., 2018). The findings of the review indicated that COA experienced four measures of HIV-related stigma, namely: enacted stigma, vicarious stigma, perceived stigma, and internalised stigma. COAs experiences of HIV-related stigma negatively influenced several domains of psychosocial well-being, including their psychological, social, and emotional well-being, self-concept and self-esteem, and future orientation. The complete methodological design and study’s findings have been published elsewhere (Liberati et al., 2009; Yassin et al., 2018). The first round of concept identification was ingrained in the meta-synthesis analysis carried out in the systematic review and acted as a literary synthesis approach towards concept development. Studies included into the review were entered into Atlas.ti – a computer-based programme used to assist in qualitative data analysis – and coded by the primary investigator (ZY). Words that appeared repeatedly and conveyed thoughts, feelings, ideas, or knowledge about HIV-related stigma and the psychosocial well-being of COA were coded. On completion of the analysis code report was generated and each code was reviewed for similarities and discrepancies as suggested by Walker and Avant (2005). A total of 72 codes signifying manifestations of HIV-related stigma and aspects of child psychosocial well-being were produced in the literary synthesis.

The results of the meta-synthesis analysis and the initial list of concepts were used to inform and facilitate the exploration of experiences and perceptions of COA regarding HIV-related stigma and their psychosocial well-being. The objective of the qualitative, exploratory, descriptive study was to explore COAs perceptions and experiences of HIV-related stigma. The in-depth exploration served to further expand and refine focal and related concepts as distilled within the systematic review. The study conducted in-depth face-to-face interviews with 13 children between of the ages of 7 and 17 years. Data were transcribed and thematically analysed. The complete methodological design and study’s findings have been published elsewhere (Yassin et al., 2019). The qualitative exploration as undertaken here allowed for a qualitative synthesis approach towards concept development. The thematic analysis undertaken in the qualitative exploration allowed for the further identification and expansion of the initial concept list. Similarly, word items were coded in Atlas.ti producing a list of codes representing concepts of HIV-related stigma and psychosocial well-being as expressed by COA. The focal concepts and related concepts extracted from the qualitative findings were consistent with those identified during the meta-synthesis analysis. However, the findings of the qualitative exploration highlighted various coping strategies and the role of social interaction and perceived social support in mediating the impact of HIV-related stigma on the psychosocial well-being of COA resulting in the addition of 34 codes.

The findings of the empiric studies (systematic review and qualitative exploratory study) were merged for further analysis. An iterative approach was implemented for the process of concept synthesis and involved the searching and clustering of words representing the phenomenon, until theoretical saturation was reached (Chinn & Kramer, 2008; Glaser & Strauss, 1967; Walker & Avant, 2005). The two concept lists generated from the empiric studies were combined to produce an extensive list of 106 initial concepts including stigmatising behaviours, domains of psychosocial well-being coping strategies and social support. The initial concepts, their associated codes and extracts were reviewed resulting in assimilation. Concepts were further refined through the process of clustering. Overlapping concepts were clustered together to form focal or overarching concepts, until theoretical saturation was reached. Focal concepts containing many sub-categories were awarded related concepts to provide clarity and structure for focal concepts.

Following assimilation and review, a total of 24 concepts were identified. These were further refined through the amalgamation of clusters of concepts that overlapped considerably resulting in a total of six focal concepts: **enacted stigma, perceived stigma, internalised stigma, coping strategies, interpersonal relations, and psychosocial well-being**. These focal concepts provided conceptual meaning and formed the basic tenets of the developed model. Additionally, horizontal themes evolving across the data set emerged during the analysis of the combined data set, contributing towards statement development. The focal concepts alongside the related concepts and horizontal themes are presented in Table 1.

**Table 1.** Focal and related concepts.

Focal concepts	Related concepts	Horizontal themes
Enacted stigma	*Overt Discrimination* *Rejection* *Exclusion* *Victimisation* *Humiliation* *Abandonment* *Unfair treatment* *Hostility* *Gossip and insults directed at deceased parent/s*	Children orphaned by AIDS reported experiences of several manifestations of enacted stigma because of parental illness and death, regardless of their own HIV status. Experiences of enacted stigma resulted in increased perceived stigma and psychological distress for children orphaned by AIDS. Reported manifestations of enacted stigma included overt discrimination, hostility, rejection, exclusion, differential treatment compared to other children within the homestead, isolation, humiliation, abandonment by extended family members, gossiping about and name calling of deceased parent, and victimisation.
Perceived stigma	*Shared perceptions of HIV-related stigma* *Beliefs about the prevalence of HIV-related stigma*	Shared perceptions of HIV-related stigma, hearing or witnessing the stigmatisation of others, and experiences of enacted stigma results in an increased perception of the prevalence and normativity of HIV-related stigma within the social environment or context of COA. As a result of perceived stigma, COA become increasingly perceptive and fearful of enacted stigma directed at them, whether real or perceived.
Internalised stigma	*Self-blame* *Shame*	Internalised stigma is presented by COA, as they internalise the negative views and beliefs of themselves as truthful and valid. These negative beliefs are incorporated into their sense of self, as COA often feel ashamed and guilty, and identify as being tainted, dirty, unlovable, unwanted, a burden or a drain on society.
Coping strategies	*Problem-focused coping* *Emotion-focused coping* *Avoidant-coping strategies*	COA utilised coping strategies to avoid experiences of HIV-related stigma and/or deal with experiences of HIV-related stigma. COA concealed their association to HIV/AIDS through a culture of non-disclosure of cause of parental death and illness (problem-focused coping) to prevent being stigmatised by others. COA engaged in positive re-appraisal, presented a strong sense of faith, assigned external attributes as the reason for stigmatisation, engaged in dis-identification with the stigmatised group and/or accepted their stigmatised social status (emotion-focused coping) to cope with experiences of stigmatisation. Children orphaned by AIDS self-isolated and withdrew themselves from others to avoid experiences of enacted stigma (avoidant-coping strategies).
Psychosocial well-being	*Psychological well-being* *Emotional well-being* *Social well-being* *Self-esteem and self-concept* *Future orientation*	COA reported increased levels of psychological distress, depression, and anxiety because of HIV-related stigma, indicating poor psychological well-being. COA are unable to healthily express their feelings and concerns regarding stressful life events. As a result, they display a negative emotional status, including feelings of sadness, anger, fear, and guilt, indicating poor emotional well-being. COA have poor interpersonal relationships. Due to avoidant-coping strategies, they are unable to form a healthy sense of intimacy with others, resulting in poor social well-being. Poor social well-being limits COAs opportunity to engage in self-esteem building actives and reappraisal through others. Additionally, internalised stigma affects the confidence, healthy self-concept and self-esteem COA, indicating lower levels of self-esteem and a poor self-concept. While COA remained optimistic about their futures, they lacked perceived control and hopefulness about their future goals and dreams. Additionally, COA lacked confidence to pursue their future goals, indicating poor future orientation.
Interpersonal relations	*Social support* *Community* *Interpersonal interaction with others*	HIV-related stigma occurs through interpersonal interaction between COA and other individuals such as family members, familial friends, and peers within a variety of social environments, for example, the homestead, community, school, etc. Through interpersonal interaction, enacted stigma and public stigma may be conveyed, increasing COAs perception of HIV-related stigma and lowering their perception of social support that they may receive from others.

#### Concept classification

Focal and related concepts distilled from the empirical phases were classified using the survey list developed by Dickoff et al. (1968) that highlights six significant activity aspects, namely: (1) agency, (2) recipiency, (3) framework, (4) dynamics, (5) procedure, and (6) terminus.

- *Agency* refers to the individual performing an activity. In this model, *agents* are non-stigmatised individuals that are neither infected nor affected by HIV/AIDS, and include, for example, family members, peers, educators, medical staff, and community members. Agents direct overt discriminatory and hostile behaviours towards COA. Enacted stigma is categorised according to agency.
- The *recipient* refers to the individual/s receiving the activity. In this model, the recipients are children under the age of 18 who have lost one (single orphan) or both (double orphan) parents to AIDS-related causes. These children experience enacted stigma and become increasingly perceptive of HIV-related stigma. Orphaned children then internalise and accept the negative view of themselves. Enacted stigma, perceived stigma, and internalised stigma are categorised in terms of recipiency.
- *Framework* is the context in which an activity is performed. *Context* in terms of this model refers to the social environment in which both the recipients and agents are present, and include the homestead, organisational care settings, school settings, and local communities. Interpersonal relations are classified in terms of framework.
- *Dynamics* is the energy or power source of the activity, which may be physical, chemical, biological, or psychological. Both agents and recipients serve as an energy and power source in this model. Agents possess physical power as they engage in acts of overt discrimination and humiliation against the recipient. The recipient possesses both physical and psychological power sources as they experience and perceive manifestations of stigmatisation and embody behavioural responses to these experiences. Enacted stigma and coping strategies are categorised in terms of dynamics.
- *Procedure* refers to general patterns, paths, or sets followed for the accomplishment of the goal. The procedure in this model unfolds within interpersonal interaction between the agent and recipient. It is through interpersonal interaction that COA may receive, experience, perceive, and respond to HIV-related stigma, resulting in poor psychosocial well-being. Interpersonal relations are categorised for procedure.
- *Terminus* or the end point of the activity is the stigmatisation and poor psychosocial well-being of COA through the process of interpersonal interaction between the agent and recipient. Through the process of interpersonal interaction between the non-stigmatised and stigmatised, measures of HIV-related stigma are directed at COA. Poor psychosocial well-being presented in COA is a cumulative result of their experience and perceptions of enacted, perceived, and internalised stigma. Psychosocial well-being is categorised in terms of terminus.

#### Defining of concepts

Adequate concepts are to be defined as they convey conceptual meaning and clarify the ideas and usages associated with the presented concepts (Chinn & Kramer, 2008; Walker & Avant, 2005). To define the concepts adequately, various sources, including dictionaries, subject textbooks, and published works were screened to fully understand the identified concepts. In this model, dictionary and subject-specific definitions were utilised to provide a synopsis of definitions contextually specific to the current model as suggested by Walker and Avant (2005). Consequentially, contextually specific definitions were created and assigned to focal concepts that were identified, categorised, and defined, and then placed in relation to each other through the development of relationship statements. An example of definitions as extracted from dictionaries, subject textbooks and published works are displayed in Table 2.^1^

**Table 2.** Definitions of concepts.

Concept	Dictionary definition	Subject-specific definition	Developed contextually specific definition
Enacted Stigma	The word enact is defined in the Collins English Dictionary as: (1) ‘to make into an act or statute’; (2) ‘to establish by law; ordain or decree’; (3) ‘to represent or perform in or as if in a play: to act out’ by the (Hanks, Long, & Urdang, 1985, p. 481). The Merriam-Webster online dictionary defines enacted as ‘to act out’. Similarly, the Oxford online dictionary defines the word enacted as ‘take place’. The Collins English Dictionary provides several definitions for the word stigma based upon various disciplines, the most applicable being: (1) ‘a distinguishable mark of social disgrace: the stigma of having been in prison’; (2) ‘any mark on the skin, such as one characteristic of a specific disease’; and (3) ‘any sign of mental deficiency or emotional upset’ (Hanks et al., 1985). Likewise, the Merriam-Webster online dictionary defines stigma as ‘a mark of shame or discredit’ and ‘an identifying mark or characteristic’ or a ‘stain’ associated with ‘a specific diagnostic sign of a disease’. The definition of stigma, as described above, is applied to the focal concepts – perceived stigma and internalised stigma – presented below.	HIV-related stigma research states that enacted stigma captures the interpersonal aspect of HIV-related stigma, which involves acts of overt discrimination and humiliation directed at individuals infected or affected by HIV/AIDS as a result of their stigmatised status (Chi et al., 2014; Wei, Li, Tu, Zhao, & Zhao, 2016). Gilbert and Walker (2010) theoretically define enacted stigma as actual cases of discrimination or discrimination by others towards individuals infected or affected by HIV/AIDS.	Enacted stigma in this study refers to the manifestation of HIV-related stigma that represents the interpersonal aspect of stigmatisation. Manifestations of enacted stigma include acts of overt discrimination, humiliation, and rejection directed at an individual because of his/her stigmatised status resulting from their association with HIV/AIDS.
Perceived Stigma	The Merriam-Webster online dictionary describes ‘perceived’ as ‘to attain awareness or understanding of’ or to ‘become aware of through the senses, especially see or observe’. The Collins English Dictionary defines perceive as ‘to become aware of (something) through the senses, esp. the sight; recognize or observe’. The Collins English Dictionary provides several definitions for the word stigma based upon various disciplines, the most applicable being (1) ‘a distinguishable mark of social disgrace: the stigma of having been in prison’; (2) ‘any mark on the skin, such as one characteristic of a specific disease’; (3) and ‘any sign of mental deficiency or emotional upset’ (Hanks et al., 1985). Likewise, The Merriam-Webster online dictionary defines stigma as ‘a mark of shame or discredit’ and ‘an identifying mark or characteristic’ or a ‘stain’ associated with ‘a specific diagnostic sign of a disease’.	In HIV-related stigma research perceived stigma captures the intrapersonal aspect of stigma and refers to ‘the subjective awareness of social stigma’ (Chi et al., 2014, p. 1055). Perceived stigma also refers to ‘all types of stigmatizing behaviours towards people living with HIV/AIDS, as perceived by themselves’ (Zhao et al., 2012, p. 276). Steward et al. (2008, p. 1226) provide an all-inclusive definition of perceived stigma which refers to ‘the subjective awareness of stigma’ which represents ‘the belief about the prevalence of stigmatizing attitudes among people in the local community, or the degree to which stigma is perceived as normative’.	In this study, perceived stigma is regarded as an individual’s subjective awareness of HIV-related stigma including their perception or belief regarding the normativity and prevalence of stigmatising beliefs, behaviours, and attitudes within the local community about HIV/AIDS and those infected and affected by HIV/AIDS
Internalised stigma	According to the Merriam-Webster dictionary, the word ***internalized*** is ‘to incorporate (values, patterns of culture, etc.) within the self as conscious or subconscious guiding principles through learning or socialization’.^a^ The Collins English Dictionary defines the word ***internalized*** as ‘to make internal, esp. to incorporate within oneself (values, attitudes, etc.) through learning or socialization’ (Hanks et al., 1985). The Collins English Dictionary provides several definitions of the word ***stigma*** based upon various disciplines, the most applicable being: (1) ‘a distinguishable mark of social disgrace: *the stigma of having been in prison*’; (2) ‘any mark on the skin, such as one characteristic of a specific disease’; and (3) ‘any sign of mental deficiency or emotional upset’ (Hanks et al., 1985). Likewise, the Merriam-Webster online dictionary defines ***stigma*** as ‘a mark of shame or discredit’ and ‘an identifying mark or characteristic’ or a ‘stain’ associated with ‘a specific diagnostic sign of a disease’.	HIV-related stigma research defines ***internalized stigma*** as ‘the extent to which an individual accepts stigma as valid’ (Steward et al., 2008, p. 3). ***Internalized stigma*** ‘involves the thoughts and behaviours stemming from the persons own negative perceptions about themselves because of their HIV status’ (Zhao et al., 2010, p. 1303). Internalised stigma, often known as self-stigma in current research, may be considered a subject-specific definition of ***internalized stigma***. *Self-stigma* is defined as the internalisation of stigma and the acceptance of its validity which is ‘manifested in negative affect toward and belief about the self’ (Herek, Saha, & Burack, 2013, p. 42).	In this study, internalised stigma refers to the internalisation of the negative and stigmatising views associated with HIV/AIDS into one’s values and beliefs about the self. In totality, ***internalized stigma*** is the acceptance of stigmatising beliefs and values by COA as truthful and valid, in turn these accepted values and beliefs are incorporated into the self.
Coping strategies	The focal concept ***coping strategies*** is divided and defined in two separate parts, namely, coping and strategies, allowing for the emergence of conceptual meaning for the current focal concept. Both the Collins English Dictionary (Hanks et al., 1985, p. 331) and the Merriam-Webster online dictionary provide the first known definition for the word ***coping*** as follows: ‘the sloping top course of a wall, usually made of masonry or brick’ and ‘the covering course of a wall usually within a sloping top’, respectively. According to the online Oxford English Dictionary, the word ***coping*** originated in the mid sixteenth century from the verb cope, originally meaning ‘dress in a *cope*’, ‘to cover’.^b^ Therefore, the definition of ‘cope’ is considered and used to define and understand the word ***coping***. *Cope* is defined as: (1) ‘to deal with and attempt to overcome problems and difficulties’; and (2) ‘to maintain a contest or combat usually on even terms or with success’.^c^ Alternatively, the Collins English Dictionary defines *cope* as: (1) ‘to contend’, and (2)’to deal successfully with or handle a situation: manage’ (Hanks et al., 1985, p. 331). ***Strategies*** is the plural of *strategy*; therefore, the word *strategy* is used to define the term ***strategies***. The Merriam-Webster online dictionary provides the following definitions for the word *strategy,* namely: (1) ‘a careful plan or method;’ (2) ‘the art of devising or employing plans or stratagems toward a goal’; and (3) ‘the adaption or complex of adaptions (as of behavior, metabolism, or structure) that serves or appears to serve an important function in achieving evolutionary success’. Similarly, the Collins English Dictionary defines *strategy* as ‘the practice or art of using stratagems’ and ‘a plan or stratagem’ (Hanks et al., 1985, p. 1437). According to the definitions presented above, the word *strategy* is closely associated with the use of the word stratagem, which is defined as ‘a cleverly contrived trick or scheme for gaining an end; skill in rues or trickery’.^d^ The Collins English Dictionary defines stratagem as ‘a plan or trick, esp. one to deceive an enemy’.	In HIV/AIDS research, ***coping strategies*** are regarded as ‘specific actions that people use to manage stress’, including ‘affective (e.g. emotional regulation), behavioural (e.g. distraction), and cognitive (e.g. remuneration) responses that people use to cope with environmental stressors’ (Chaudoir et al., 2012, p. 2384). Additionally, coping strategies or processes are ‘typically characterized as relatively stable, individual difference measures that characterize the general strategies that people use to cope with stressors’ (Chaudoir et al., 2012, p. 2384).	In this study, ***coping strategies*** refer to specific actions that are stable over time and involves the complex adaption of affective, behavioural, and cognitive responses of an individual to successfully deal with stressors, in this case being the experiences and perceptions of HIV-related stigma.
Interpersonal Relations	The word ***interpersonal*** is simply defined by the Merriam-Webster online dictionary as ‘being, related to, or involving relations between people’.^e^ Similarly, the online Oxford Dictionary defines ***interpersonal*** as ‘relating to relationships or communication between people’.^f^ The Collins English Dictionary defines ***relations*** as: (1) ‘social, political, or personal connections or dealing between or among individuals, groups, nations, etc.’, and (2) ‘family or relatives’ (Hanks et al., 1985, p. 1232). Furthermore, the Merriam-Webster online dictionary defines ***relations*** as: (1) ‘the way in which two or more people or things are connected; a things effect on or relevance to another’; (2) ‘the way in which two or more people or groups feel about and behave towards each other’; (3) and ‘a person who is connected by blood or marriage; a relative’.^g^	According to the psychology of ***interpersonal relations*** discussed by Heider (2013, p. 1), interpersonal relations ‘denotes relations between a few, usually two people. How one person thinks and feels about another person, how he perceives him and what he does to him, what he expects him to do or think, and how he reacts to the actions of the other’.	In this study, the focal concept of ***interpersonal relations*** refers to the social interaction between the stigmatised – children orphaned by AIDS – and their social environment with other individuals. This includes the stigmatised relationship and interaction with surviving relatives, extended family, friends, and the surrounding community or others present within their social environment. Additionally, in the context of this study ***interpersonal relations*** include the stigmatised interaction with non-stigmatised groups of individuals and their perception of others, including their perception of social support offered by others.
Psychosocial well-being	The focal concept is defined in two separate parts to give rise to conceptual meaning, namely, ***psychosocial*** and ***well-being,*** as described below. The Oxford Online Dictionary defines ***psychosocial*** as ‘of or relating to processes or factors that are both social and psychological in origin’ (Hanks et al., 1985, p. 1179). The Merriam-Webster online dictionary similarly defines ***psychosocial*** as ‘involving both psychological and social aspects; relating social conditions to mental health’. The Oxford English Dictionary expands on the previously provided definitions, defining the word ***psychosocial*** as ‘relating to the interrelation of social factors and individual thought and behavior’. The Collins English Dictionary defines ***well-being*** as ‘the condition of being contended, healthy, or successful: welfare’ (Hanks et al., 1985; p. 1645). Similarly, the Merriam-Webster online dictionary and the Oxford Online English dictionary defines ***well-being*** as ‘a state of being happy, healthy, or prosperous: welfare’ and ‘a state of being comfortable, healthy, happy’, respectively.^h^ The word ***well-being*** is unanimously defined as being ‘healthy’ by all sources as presented in the definitions provided above (Hanks et al., 1985; the Oxford Online English dictionary; Merriam- Webster online dictionary). The Collins English Dictionary defines the word ‘healthy’ as ‘enjoying good health; functioning well or being sound’ (Hanks et al., 1985, p. 676). The Merriam-Webster online dictionary expands on the definition of healthy as ‘free from disease or pain: enjoying healthy and vigor of body, mind, or spirit; showing physical, mental or emotional well-being: evincing health’.^i^	The term ***psychosocial well-being*** is generally regarded as the ‘physical, psychological, social and cognitive well-being’ of an individual (Wang, He, & Dadialla, 2014, p. 23). While there remains little consensus among scholars for defining the term psychosocial well-being, a holistic definition was provided by Fujishima-Hachiya and Inoue (2012), who define psychosocial well-being as the amalgamation of psychological and social well-being, encompassing well-being to include social inclusion and resilience, that reflects an individual’s thoughts, behaviours and reactions to the social environment. This conceptual definition is consistent with the measures used in several studies focusing on orphaned children and HIV/AIDS to determine psychosocial well-being and the related concepts identified following the findings of the current study.	In summary, psychosocial well-being in this study refers to the holistic, healthy functioning and well-being of children who have been orphaned by AIDS, encompassing their psychological well-being to include psychological, social, and emotional well-being that reflects their thoughts, behaviours, and their reactions to their social environments. Psychosocial well-being in this regard consists of five crucial domains, namely, psychological well-being, emotional well-being, social well-being, self-concept and self-esteem, and future orientation, comprising the holistic well-being of a given individual.

^a^ ‘Internalize', Merriam-Webster Dictionary [Online]. Viewed from https://www.merriam-webster.com.

^b^ ‘Cope’, Oxford English Dictionary. Viewed from https://www.lexico.com/en/definition/coping.

^c^ ‘Cope’, Merriam-Webster Dictionary [Online]. Viewed from https://www.merriam-webster.com.

^d^ ‘Stratagem', Merriam-Webster Dictionary [Online]. Viewed from https://www.merriam-webster.com.

^e^ ‘Interpersonal', Merriam-Webster Dictionary [Online]. Viewed from https://www.merriam-webster.com.

^f^ ‘Interpersonal’, Oxford Dictionary [Online]. Viewed from https://www.lexico.com.

^g^ ‘Relations’, Merriam-Webster Dictionary [Online]. Viewed from https://www.merriam-webster.com.

^h^ ‘Well-being', Merriam-Webster Dictionary [Online]. Viewed from www.merriam-webster.com; ‘Well-being', Oxford English Dictionary. Viewed from https://www.lexico.com/definition/well-being.

^i^ ‘Healthy’, Merriam-Webster Dictionary [Online]. Viewed from www.merriam-webster.com.

### Statement development

Statements serve to specify the relationship/s between two or more concepts to provide the structure that is central to the developed model (Walker & Avant, 2005). Furthermore, developed statements provide the ability to describe, predict, and explain the nature of the interactions between the focal concepts and are guided by the question, ‘what is the nature of the relationships?’ (Chinn & Kramer, 2008, p. 86). As statements may be derived from quantitative, qualitative, and literary methods, as proposed by Walker and Avant (2005), this study utilises a statement synthesis strategy that derives relationship statements from evidence emerging from the systematic review and qualitative exploration in combination with the survey list utilised for the categorisation of concepts. As suggested by Walker and Avant (2005), the meta-synthesis and thematic analysis carried out within the empiric phases allowed for the identification of relationships among focal and related concepts. Further analysis allowed for the logical movement from evidence to inferences or conclusions conveyed by a set of inter-related studies. The themes and corresponding evidence for both empiric studies were combined and reviewed by two investigators (ZY, CE) in order extract patterns of relationships between the identified concepts. Placing concepts in relation to each other through the use of a survey list by Dickoff et al. (1968), assisted investigators with the identification of relationships among concepts. Once a catalogue of relationships between variables as reported in the literature or expressed by participants in the qualitative exploration was developed, the investigators developed and documented general inferences regarding the relationships between focal and related concepts. The relationship statements of the developed model are as follows:

- COA experience enacted, perceived, and internalised stigma. These experiences are facilitated by the stigmatised child’s interpersonal relations with other individuals within their social environment.
- There is a bidirectional relationship between enacted and perceived stigma. When children encounter enacted stigma, their experience of perceived stigma – the perception of the normativity and prevalence of stigmatisation associated with HIV/AIDS – is heightened. Perceived stigma is accompanied by increased enacted stigma, as the stigmatised child anticipates the experience of enacted stigma and becomes increasingly sensitive to the actions of others. Enacted and perceived stigma leads to internalised stigma – the incorporation of negative beliefs and views about the self that are regarded as valid and truthful by COA.
- Poor psychosocial well-being is the direct consequence of enacted and internalised stigma. Enacted stigma negatively affects the domains of psychological and social well-being. Internalised stigma has detrimental outcomes for the domains of self-concept and self-esteem, and future orientation.
- In the absence of social support, negative outcomes associated with enacted, perceived, and internalised stigma are mediated by maladaptive coping strategies adopted by COA. These coping strategies are adopted to minimise and deal with the consequences of HIV-related stigma or avoid future experiences of enacted stigma.
- Poor psychosocial well-being is the accumulative outcome of enacted stigma, perceived stigma, experienced internalised stigma, and coping strategies adopted by COA.
- The psychosocial well-being of COA is comprised of five interconnected domains of well-being, which are negatively and uniquely affected by each other.

### Model description

This description of the model provides an overview and centres around the six descriptive components proposed by Chinn and Kramer (2008), namely: (1) purpose, (2) concepts, (3) definitions, (4) relationships, (5) structure, and (6) assumptions of the social transactional model of HIV-related stigma and the psychosocial well-being of children orphaned by AIDS. The six key questions identified by Chinn and Kramer (2008) further guided the description of the model, which facilitates a clear understanding of the nature and flow of the model for use in research and practice (Chinn & Kramer, 2008).

#### Overview of the model

**Figure 1.**
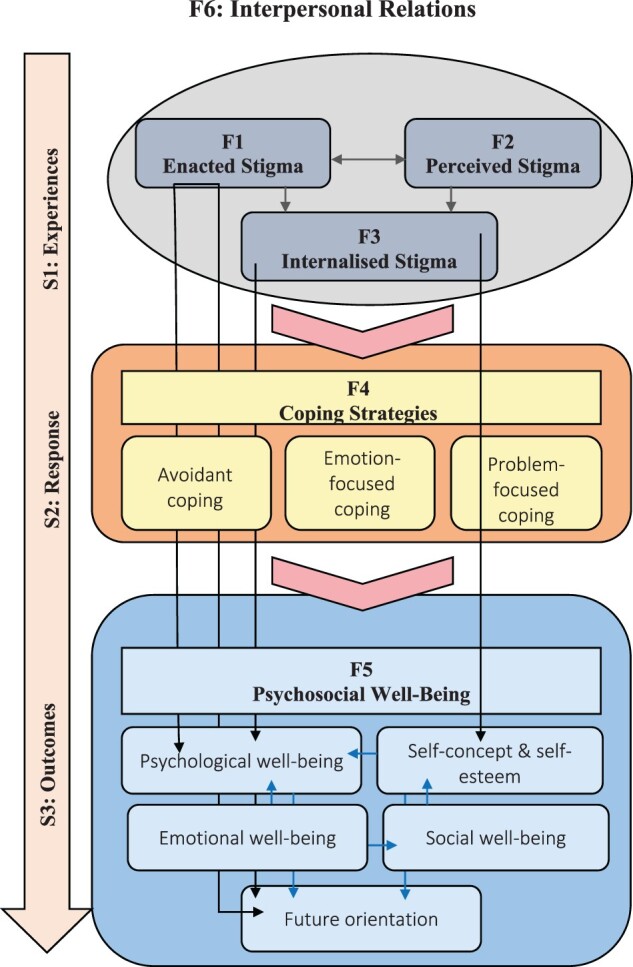
The social transactional model of HIV-related stigma and the psychosocial well-being of COA.

The model serves as a framework for understanding the manner in which HIV-related stigma affects the psychosocial well-being of COA. Both interpersonal (enacted stigma) and intrapersonal (perceived and internalised stigma) measures of HIV-related stigma varied in the way they affected the psychosocial well-being of COA. The experiences and perceptions of HIV-related stigma led to their use of maladaptive coping strategies – a mediator contributing to the poor psychosocial well-being exhibited by COA. Maladaptive coping strategies simultaneously provide protection against and relief of experiences of stigmatisation experienced by COA, while negatively affecting their psychosocial well-being. The model captures five core domains of psychosocial well-being, holistically encompassing the poor psychosocial well-being exhibited and described by COA. It is viewed as a social interactional model as it denotes that a child’s interactions with their social environment (friends, family, and community) shapes their behaviour, development, and well-being. Therefore, the social transactional model of HIV-related stigma and the psychosocial well-being of COA consisted of three stages in which HIV-related stigma acts to affect psychosocial well-being, namely (1) experiences, (2) responses, (3) and outcomes. A graphical representation of the social transactional model of HIV-related stigma and the psychosocial well-being of COA is presented in Figure 1.

#### Purpose of the model

Watson (1985) proposes that theory is generated to enhance the understanding of a given phenomenon. Aligned with Watson (1985), this model serves a theoretic purpose as it provides a framework that enhances the understanding of the manner in which HIV-related stigma affects the psychosocial well-being of COA. The model fosters an understanding of two sources of phenomenon and its relation to one another – related stigma and the psychosocial well-being of COA.

#### Context of the model

The *context* of the model is any social environment in which COA are present, such as the homestead, school, health facilities, and local and surrounding communities. While the model was developed within the South African context, it was based on national and international empirical findings. From a cultural perspective, the model may be applicable in various contexts, which includes individuals from diverse ethnic and cultural backgrounds.

#### Assumptions of the model

*Assumptions* of the model are regarded as the basic givens or truths that are accepted and assumed to be factual (Chinn & Kramer, 2008), and that influence the structure and conceptualisation of the model (Chinn & Kramer, 2008). The assumptions of the model presented below were drawn from a developed conceptual framework. A literature search was carried out to identify existing theories, conceptual understanding and published works exploring HIV-related stigma or/and child well-being. These conceptual underpinnings were critically evaluated to develop basic assumptions regarding the nature and characteristics of the studied phenomenon. The assumptions of the social transactional model of HIV-related stigma and the psychosocial well-being of COA are described below:

- Stigmatisation is a social construct embedded within the social environment of COA and is dependent on the process of dyadic social interaction occurring between COA and non-stigmatised individuals. It is through dyadic social interaction that the cultural and social understandings of HIV-related stigma are conveyed and enacted.
- COA acutely experience HIV-related stigma as a result of their association with parental HIV/AIDS and are awarded the same discredited identity as their parents.
- COA experience both interpersonal and intrapersonal forms of stigma. Interpersonal forms of HIV-related stigma are represented by social interaction between COA and others that is characterised by discrimination, rejection, and hostility. Intrapersonal forms of HIV-related stigma represent COAs psychological and internal experience and response to HIV-related stigma.
- COA experience various components of HIV-related stigma that are both interrelated and interdependent, including enacted stigma, perceived stigma, and internalised stigma.
- Each component of HIV-related stigma uniquely contributes to poor psychosocial well-being and psychosocial distress among COA. These components of HIV-related stigma may, directly or indirectly, via maladaptive coping strategies, negatively affect the psychosocial well-being of COA.
- The various domains of psychosocial well-being of COA, such as the psychological-, social- and emotional well-being, self-esteem and self-concept, and future orientation, are affected.
- Domains of psychosocial well-being are codependent and interrelated, as a change in one domain results in a change in another, resulting in overall poor psychosocial well-being.

#### Definitions of key concepts

Definitions have been developed to provide conceptual meaning and clarity to focal concepts which formed the basic building blocks of the social transactional model of HIV-related stigma and the psychosocial well-being of COA.

- **Enacted stigma**
- *Enacted stigma* within the context of this model refers to the interpersonal aspect of stigmatisation as it occurs within the social interaction or communication between stigmatised and non-stigmatised COA and other individuals within their social environment. Enacted stigma is regarded as the experience of overt discrimination, humiliation, and rejection of an individual because of his/her devalued status resulting from their association with HIV/AIDS.
- **Perceived stigma**
- *Perceived stigma* refers to the intrapersonal aspect of stigmatisation drawing on the perceptive reality of an individual, thus being regarded as an individual’s subjective awareness of HIV-related stigma. This awareness includes their perceptions and beliefs regarding the normativity and prevalence of stigmatising beliefs, behaviours, and attitudes held by the community about HIV/AIDS and those infected and affected by HIV/AIDS. In essence, perceived stigma presents a stigmatised individual’s expectation or anticipation of experiencing stigmatisation from others.
- **Internalised stigma**
- *Internalised* stigma, also commonly known as self-stigma, is the intrapersonal aspect of stigmatisation. Internalised stigma denotes the acceptance of stigmatising beliefs and values, which are regarded as truthful and valid by stigmatised individuals, in this case, COA. These accepted beliefs and values are incorporated into the self and manifested as feelings of guilt, shame, unworthiness, and the view of oneself as being unworthy, dirty, or a drain on society. Two main related concepts – self-blame and shame – are also defined. Janoff-Bulman’s (1979) conceptualisation of characterological self-blame, which is esteem related, serves to offer an adequate conceptualisation of the related concept ‘self-blame’. Here, self-blame is attributed to one’s character and is associated with one’s belief that past negative outcomes are a result of personal deservingness. The related concept *‘*shame' represents an internal state of regret, dishonour, or inadequacy. COA often express shame because of their devalued social status and being associated with HIV/AIDS, which is regarded as taboo within social and cultural settings. The experience of internalised stigma, self-blame, and shame is evidenced by a poor sense of self.
- **Coping strategies**
- *Coping strategies* is a process whereby stigmatised individuals adopt complex affective, behavioural, and cognitive responses to successfully deal with stressors and negative life experiences. The manner in which stigmatised individuals adopt these coping strategies are relatively stable and consistent over time. Stigmatised individuals attempt to mitigate the negative impact of HIV-related stigma through the adoption of various coping strategies. These coping strategies are identified as the related concepts of: (1) emotion-focused coping, (2) avoidant-coping strategies, and (3) problem-focused coping. *Problem-focused coping* strategies seek to alter the relationship between stigmatised individuals and their social environment and may be directed at oneself, others, or the presented situation. Generally, these strategies are aimed at problem solving and may include non or selective disclosure, compensation, activism, social support, or disengagement. Alternatively, *emotion-focused coping* refers to strategies that seek to regulate negative emotions and strive towards reducing or managing the emotional distress accompanied by stressful situations or cues. These strategies include downward spiral comparisons and external attributions for stigmatising behaviours, like the ignorance and denial of others, dis-identification, and the positive reappraisal of experiences. Lastly, *avoidant-coping* refers to the cognitive and behavioural efforts of the stigmatised individuals to avoid or minimalise dealing with stressors and negative situations, such as withdrawal or isolation.
- **Interpersonal relations**
- *Interpersonal relations* are the social environments in which stigmatised individuals exist and function. In this sense, interpersonal relations extends beyond the social environment in which the individual is present to include social interaction between the stigmatised individual and non-stigmatised individuals, such as family, friends, community members, service providers, and others present within the social environment. Through social interaction in the social environment, HIV-related stigma is extended, directed at, or conveyed to COA.
- **Psychosocial well-being**
- *Psychosocial well-being* is the holistic functioning and well-being of a child, which encompasses the domain of psychological well-being and includes social and emotional well-being, self-esteem and self-concept, and future orientation, which reflects the thoughts, behaviour, and an individual’s reactions towards their social environment. Five domains of psychosocial well-being were defined for a comprehensive understanding of the focal concept as applied within this study. *Psychological well-being* is a multidimensional concept that centres on the mental health or state of an individual, and is developed through life experiences, personal identity, and emotional regulation. In the context of this study, poor psychological well-being is represented by depression, anxiety, and post-traumatic stress. *Emotional well-being* is closely related to psychological well-being and refers to positive or negative effects. In the context of this study, emotional well-being refers to the emotional affect of the stigmatised individual, which is negatively affected by psychological distress. Poor *emotional well-being*, in this study, is detected by the presence of poor emotional regulation and a negative emotional status presented by the stigmatised individual. *Self-concept* and *self-esteem,* affected by emotional well-being, presents a way to think about oneself, and refers to the conscious reflection of an individual regarding their being or identity that remains separate from the environment and others. *Self-concept* refers to a multifaceted system of learned beliefs, attitudes, and opinions, which are regarded as true by the stigmatised individual about their personal existence. Self-concept is associated with *self-esteem*, which presents the affective or emotional aspect of the self, or the way an individual feels about and values themselves. In the context of the study, individuals are mutually dependent upon their social environment; therefore, *social well-being* refers to social inclusion, a sense of belonging, and the interpersonal relationships held with others. Stigmatised individuals who are socially excluded, rejected, and unable to form healthy interpersonal relationships with others, display poor social well-being. Lastly, the related concept *future orientation* broadly refers to the extent that an individual thinks about their future and includes optimism, hopefulness, and perceived control over the future.

#### Model structure

The structure of the social transactional model of HIV-related stigma and the psychosocial well-being of COA consists of three linear stages according to the identified focal concepts, which facilitates a discussion and explanation of the interaction between focal concepts and stages of the model. The description of the structure of the model as presented here corresponds with the graphical representation of the model presented in Figure 1.

The stages of the model are illustrated by the letter ‘S’ to represent the word ‘stage’, i.e. stage one is presented as ‘S1: Experiences’. The numbering of the stages is significant as it depicts the linear structure of the model, while their positioning within a vertical downward pointing arrow suggests movement from HIV-related stigma to psychosocial well-being, indicating the linear progression of the stages. Focal concepts are depicted by the letter ‘F’, such as ‘F1’ for enacted stigma. Unlike the stages presented in the model, the numbering of focal concepts holds no significance, as concepts are rather iterative, involving bidirectional and linear relationships. Focal concepts are presented in bold text to draw attention to the importance of main concepts, setting them aside from related concepts. Related concepts attached to focal concepts that facilitate understanding of the model are included in plain italicised text underneath the presented focal concepts.

**Interpersonal relations (F6)** encapsulated the experiences, responses, and psychosocial outcomes for COA. Interpersonal relations represent the social environment in which COA exist and function, affecting them throughout the three stages of the model. The social environment in the context of this study comprises the homestead, school, community, health care facilities, and local organisations. For the process of stigmatisation to unfold, social interaction between the stigmatised child and non-stigmatised individuals – referred to as mixed contact in the social environment – needs to be present (Goffman, 1963). Forms of mixed contact would include interaction between stigmatised orphaned child and family members, friends, children within the surrounding community, educational staff, community members, health service providers, and organisational staff. While it is envisioned that interpersonal relations would be present throughout the model, it is most influential during stage one as stigmatisation is extended to or perceived by COA through mixed contact. Interpersonal relations, or rather the absence thereof, perpetuates poor psychosocial well-being and the adoption of maladaptive coping strategies by COA.

## Stage one: experiences

During stage one (S1), COA experience both interpersonal and intrapersonal forms of stigmatisation, conveyed through the process of social interaction within their social environment. **Enacted stigma (F1),** an interpersonal form of HIV-related stigma, is characterised by overt discrimination and hostility resulting in social exclusion, placing COA at a disadvantage (Major & O'Brien, 2005). Manifestations experienced by COA include rejection, hostility, exclusion, ill or unequal treatment, devaluation, victimisation, exploitation, and abuse. There is a direct relationship between **enacted stigma (F1), psychosocial well-being (F5)** and intrapersonal forms (F2, F3) of stigma. Firstly, the direct relationship between enacted stigma and psychosocial well-being is presented by black vertical arrows pointing towards the domains of **psychosocial well-being (F5)** that are negatively affected. Experiences of enacted stigma inhibit the *psychological well-being* of COA, increasing their levels of distress and the formation of psychopathological symptoms, such as depression, anxiety, adjustment problems, post-traumatic stress, fear, and distress. Enacted stigma leads to the obstruction of formal education and educational access. Such obstruction coupled with the presence of overt discrimination within the schooling environment, negatively affects future orientation, as COA present a lack of optimism, confidence, and perceived control over their future.

There is a bidirectional relationship between **enacted (F1)** and **perceived (F2)** stigma as presented by a double arrow between the two focal concepts. Firstly, as children experience enacted stigma, they become increasingly aware of HIV-related stigma. This contributes to the development of **perceived stigma (F2),** which is regarded as the belief about the prevalence and normativity of HIV-related stigma within one’s environment. Secondly, psychopathological symptoms ensued from enacted stigma predicts perceptions of discrimination – perceived stigma (Major & O'Brien, 2005) – as children with internalising problems are increasingly sensitive and perceptive to stigmatising behaviours, increasing experiences of enacted stigma over time. Perceived stigma fails to directly affect the psychosocial well-being of COA, but like enacted stigma contributes to the development of **internalised stigma (F3).** The arrows pointing towards internalised stigma (F3) from enacted and perceived stigma indicate this relationship.

**Internalised stigma (F3),** a product of enacted and perceived stigma, is the internalised belief of being devalued and tainted and results in feelings of self-hatred, guilt, inferiority, as well as shame and embarrassment for COA. Internalised stigma inhibits psychosocial well-being, specifically the *self-concept* and *self-esteem,* and *psychological well-being* of COA. COA hold a negative view of themselves and lack self-esteem and confidence because of their belief that they are devalued, consequentially resulting in distress and psychopathological symptoms.

## Stage two: response

Stage two (S2) focuses on COAs response to HIV-related stigma and its related distress and negative affect. Experiences of HIV-related stigma are followed by **coping strategies (F4)** and are presented by a horizontal downward chevron. ‘Coping strategies’ is labelled ‘F4’ and has three related concepts: (1) *avoidant-coping*, (2) *emotion-focused coping,* and (3) *problem-focused coping,* which are represented by three smaller rounded rectangles. These related concepts are illustrated by smaller yellow boxes and are encapsulated by the focal concept ‘coping strategies’ (F4). **Coping strategies (F4)** include behavioural and psychological responses to stressful events, such as interpersonal and intrapersonal forms of stigmatisation, and are used either alone or in combination. Both avoidant and problem-focused coping strategies are behavioural responses to HIV-related stigma and affects psychosocial well-being. *Avoidant-coping strategies* involve disengagement, presenting the social withdrawal and self-isolation of COA to avoid experiences of enacted stigma. Disengagement exacerbates psychological distress, bringing about loneliness and actively undermines the healthy development of meaningful social relationships and networks (Schibalski et al., 2017). Problem-focused coping, which is fuelled by perceived stigma, involves secrecy, selective disclosure, and non-disclosure of the cause of parental bereavement (Stutterheim et al., 2012). In addition, these coping strategies prohibit the formation of healthy relationships and reduce the opportunity for COA to healthily express their feelings and discuss stressful life events. *Emotion-focused coping* is a psychological response to HIV-related stigma and aims to reduce negative emotional responses to stigma. COA are rather avoidant of healthily expressing their feelings and suppress the negative feelings associated with enacted, perceived, and internalised stigma. The coping strategies adopted by COA are rather maladaptive and despite their use for defending against HIV-related stigma and emotional responses, negatively affect their psychosocial well-being. The impact of coping strategies on psychosocial well-being is illustrated by a horizontal downward chevron representing the progression from coping strategies towards the psychosocial well-being of COA.

## Stage three: psychosocial well-being

Stage three (S3) presents the progression of COAs experiences and responses of HIV-related stigma to its associated outcomes for psychosocial well-being, presented by a horizontal downward chevron. Through experiences of HIV-related stigma and the adoption of maladaptive coping strategies, the **psychosocial well-being (F5)** of COA are negatively affected. Psychosocial well-being is regarded as the holistic well-being of COA, which included their psychological, emotional and social well-being, self-esteem and self-concept, and future orientation. These domains of psychosocial well-being reflect COAs thoughts, behaviours and reactions towards their social environment and others. Psychosocial well-being is presented by a rounded rectangle presenting the holistic psychosocial well-being of a COA. The rounded rectangle consists of a smaller regular rectangle set above five smaller rounded rectangles. The smaller regular rectangle presents the focal concept ‘**psychosocial well-being’ (F5),** while the smaller rounded rectangles below present related concepts forming the psychosocial well-being, namely, *psychological well-being, social well-being, emotional well-being, self-concept and self-esteem,* and *future orientation.* The order in which related concepts are presented hold no relevance as the dimensions are interconnected, related, and influence each other. The relationship between the related domains of psychosocial well-being are indicated through the use of blue arrows pointing in the direction of the affected domain.

**Enacted stigma (F1)** negatively affects the psychosocial well-being of COA. Experiences of enacted stigma acts to increase psychological distress, contributing to the development of internalising and externalising disorders, such as anxiety, depression, PTSD, and aggressive and risk-taking behaviours. The self-isolation and social withdrawal of COA (*avoidant-coping*) to prevent the future experience of HIV-related stigma contributes to the psychological distress and suffering of these children, exacerbating poor *psychological well-being*. Similarly, the *future orientation* of COA is negatively affected by the disadvantage cultivated by **enacted stigma (F1)**, manifesting as actions of overt discrimination and rejection. COA are stripped of their resources, treated unfairly, and discriminated against, both within the homestead and the schooling environment, resulting in school dropout. Without scholastic knowledge and social support, a child orphaned by AIDS lacks optimism, confidence, and perceived control over their future. There is also a direct relationship between *psychological well-being* and *future orientation,* presented by a solid blue arrow leading from psychological well-being towards future orientation. Poor psychological well-being leads to poor future orientation for COA.

**Perceived stigma (F2)** causes a child orphaned by AIDS to embody a culture of secrecy, non-disclosure, or selective disclosure of the cause of parental death. The use of *problem-focused coping strategies* to conceal the cause of parental death actively limits a child’s ability to form healthy interpersonal relationships, which are essential for receiving the social support necessary to cope with stressful life events. Therefore, perceived stigma indirectly negatively affects the *social well-being* of COA through their use of *problem-focused coping*. A direct relationship exists between *social well-being*, *self-concept and self-esteem*, and is presented by a solid blue arrow. Poor *social well-being* exhibited by a child orphaned by AIDS restricts their opportunity to engage in self-esteem enhancing activities that occurs through their social interaction with others. Through minimal interpersonal engagement, COA cannot receive self-validation from others, resulting in a poor *self-concept* and lower levels of *self-esteem* as they lose confidence in themselves and their capabilities. Non-disclosure and secrecy prohibits an orphaned child from healthily expressing their emotions and concerns about stressful events, contributing to poor *emotional well-being*.

S*elf-esteem and* the *self-concept* are similarly affected by **internalised stigma (F3)**. COA hold a negative view of themselves as being devalued, tainted, and dirty. This not only presents the poor self-concept embodied by COA, but also represents their psychological suffering. A solid blue arrow signifies the relationship between *poor self-concept and self-esteem,* and *psychological well-being*. A poor self-esteem cultivates a lack of confidence in a stigmatised child, ultimately negatively influencing their *future orientation*.

Consequentially, all forms of HIV-related stigma results in emotional distress for COA, negatively affecting their *emotional well-being*. To efficiently deal with and minimise emotional distress, COA adopt *emotion-focused coping strategies* likely to hinder healthy emotional regulation and reinforce a negative emotional status. In the absence of social support and healthy interpersonal relationships combined with experiences and perceptions of HIV-related stigma, these children are unable to healthily express themselves and continue to suppress their emotions, leading to poor emotional regulation and a negative emotional status, indicating poor *emotional well-being*. *Emotional well-being* directly affects *psychological well-being,* perpetuating psychological distress and internalising psychopathological symptoms. A solid blue arrow directed towards psychological well-being indicates this direct relationship.

### Model evaluation

A modified Delphi technique was implemented to assess the functionality of the social transactional model of HIV-related stigma and the psychosocial well-being of COA. The functionality of the model was assessed according to the points of critical reflection outlined by Chinn and Kramer (2008), including (1) simplicity, (2) clarity, (3) generalisability, (4) accessibility, and (5) importance. These points seek to contribute to an understanding of how well a developed model relates to practice, research, and/or educational activities (Chinn & Kramer, 2008). Non-probability purposive and snowball sampling techniques were utilised to identify and select a sample population consisting of knowledgeable key stakeholders as suggested by Duffield (1993). Participants were required to be (1) healthcare professionals; (2) organisational members; (3) scholars focusing on stigma, discrimination, and child well-being; (4) renowned experts in the field; and (5) informal caregivers (Hsu & Sandford, 2007). Key stakeholders deemed meeting the selection criteria was invited to participate in the study. A total of 35 identified panel experts were invited to participate in the study. These potential panel experts were geographically diverse and held varying roles and expertise within the field of interest, allowing for the recruitment of a heterogenous sample.

The invitation furnished participants with an information sheet outlining the purpose, benefits and risks of the study. Participating key stakeholders were required to provide their informed consent via email, aligned to the quality indicator proposed by Boulkedid, Abdoul, Loustau, Sibony, and Alberti (2011). Participants were informed of their rights to anonymity, confidentiality, and withdrawal from participation at any time without any repercussions. Aligned with the indicators of quality, the selected panel of experts were heterogenous in nature, speaking to the creditability and acceptance of quality indicators as the panel reflects a full range of stakeholders who are interested in the developed model and the empirical evidence upon which it is based. The diverse views of the panel of experts enriched the results of the Delphi procedure (Boulkedid et al., 2011).

During the first round of the study, key stakeholders were provided with a self-administered questionnaire via email. The self-administered questionnaire consisted of three sections: (A) a full description of the developed model, (B) biographical information, and (C) a 30-item adaptation of the model evaluative guide provided by Chinn and Kramer (2008). For section A, a full narrative description which outlines the structural process, concepts, and relationship statements of the developed model was provided. In Section B, the following biographical information was elicited from the panel of experts, namely, demographic details, gender, institution, occupation, and area of interest. Section C presented 30-items pertaining to the (1) clarity, (2) simplicity, (3) generalisability, (4) accessibility, and (5) importance of the social transactional model of HIV-related stigma for child psychosocial well-being as suggested by Chinn and Kramer (2008). Items were rated using a 4-point Likert scale ranging from (1) Strongly Disagree, (2) Disagree, (3) Agree, and (4) Strongly Agree to determine the consensus among participating panel experts.

Data were subject to a process of data analysis in which a descriptive statistical analysis was carried out using SPSS (version 26). Raw data were checked for completeness and correctness prior to analysis. Aligned with quality indicators descriptive statistics and levels of dispersion are commonly used to present collective judgements of responding experts (Hasson, Keeney, & McKenna, 2000). In adherence with these guidelines, the 4-point Likert scale was dichotomised into two categorical sections namely: (1) non-consensus, and (2) consensus. Non-consensus comprises the ratings ‘Strongly Disagree’ and ‘Disagree’, while consensus comprises the ratings ‘Agree’ and ‘Strongly Agree’. For consensus to be reached, collective ratings within a dichotomised category need to account to 80% or more of collective responses (Yeh, Van Hoof, & Fischer, 2016). The median – required to be 3.24 or higher – was calculated to determine expert consensus and is strongly favoured as it inherently appears to best reflect the convergence of opinion (Hill & Fowles, 1975; Hsu & Sandford, 2007). Additionally, levels of dispersion – the interquartile range – was identified and calculated to reflect respondent consensus.

#### Biographical information

Fourteen experts indicating a 40% response rate critically evaluated the model. The majority of participants (78.57%) resided in South Africa, followed by Zimbabwe (7.14%), England (7.14%), and Dubai (7.14%). Eight participants (57.14%) were male with the remaining six participants (42.86%) being female. More than half of the participants (64.29%) indicated that they had a background in psychology, followed by backgrounds in social work (28.57%) and nursing (7.14%). Participants (57.14%) were academic staff members at institutions of higher education, directors (14.28%) of university centres, and practitioners (14.28%) (one was a psychologist and another a social worker). The remaining two participants were an organisational member and a postgraduate student, respectively. While participants’ occupations remained heterogeneous in nature, they unanimously maintained a key interest in health-related stigma, HIV/AIDS, and child well-being.

#### Clarity

Six items relating to clarity received the highest agreement indicated by a median score ranging from 3.50 to 4. Key stakeholders (100%) indicated that (1) the view of the person and the environment are compatible, and (2) the focal concepts and relationship statements of the model were both identifiable and explicit. All participating experts (100%) supported the definitions ascribed to focal concepts, while the majority of experts (92.85%) agreed that the model is easily comprehensible. Results relating to clarity are presented in Table 3.^2^

**Table 3.** Clarity.

Item	Statement	Range	Consensus	Mean	Median	Interquartile Range
1	Focal concepts of the model are made explicit and identifiable.	3–4	100	3.80	3.50	1
2	Focal concepts within the model are well defined.	3–4	100	3.64	4	1
3	Definitions of concepts in the model are specific and applicable.	3–4	100	3.50	3.50	1
4	The explanation of concepts is appropriate and useful.	3–4	100	3.64	4	1
5	The view of person and environment is compatible.	3–4	100	3.43	3	1
6	Relationships within the model are identifiable.	3–4	100	3.57	4	1
7	All relationships are appropriate and fit within the model.	3–4	100	3.43	3	1
8	The order of the model is easily comprehended.	2–4	92.85	3.43	3.50	1

#### Simplicity

Simplicity of the developed model was indicated by a consensus rate ranging from 85.71 to 100%. Two items relating to the differentiation of concepts and the purpose of the model attained the highest agreement indicated by a median score of 4. All participating experts (100%) agreed that the model is aligned with its purpose to describe and explain phenomenon and that concepts may be differentiated or merged without the loss of theoretic meaning. While the participating experts agreed on the organisation of relationship statements within the model, only 92.85% indicated that such relationships are easily identified. Results relating to simplicity are presented in Table 4.

**Table 4.** Simplicity.

Item	Statement	Range	Consensus	Mean	Median	Interquartile Range
1	The relationships within the model are easily identified.	2–4	92.85	3.21	3	1
2	The relationships within the model are organised.	3–4	100	3.36	3	1
3	Concepts are differentiated into focal concepts and related concepts.	3–4	100	3.71	4	1
4	Concepts can be combined without losing theoretic meaning.	3–4	100	3.43	3	1
5	The model is simplistic and fosters a clear understanding throughout.	2–4	85.71	3.29	3	1
6	The model aims to describe, explain, or predict phenomenon.	3–4	100	3.57	4	1

#### Generalisability

Generalisability received the highest level of agreement across all domains, demonstrated by a median score of 4 and a 100% consensus rate across all items pertaining to the domain. All the participating experts (100%) strongly agreed that the (1) purpose of the model was clear and specific regarding its application; (2) concepts of HIV-related stigma may be applied meaningfully as presented within the developed model, and (3) that the model is both specific to areas of health-related stigma and child well-being (Table 5).

**Table 5.** Generalisability.

Item	Statement	Range	Consensus	Mean	Median	Interquartile Range
1	The purpose of the model is clear and specific.	3–4	100	3.64	4	1
2	The model can be applied to all practice areas dealing with HIV-related stigma and child psychosocial well-being.	3–4	100	3.57	4	1
3	The model is specific to the interest area of health-related stigma and child well-being.	3–4	100	3.57	4	1
4	A wide range of professionals and researchers may use the developed model.	3–4	100	3.57	4	1
5	Concepts of the model may be meaningfully applied.	3–4	100	3.71	4	1

#### Accessibility

While receiving 100% consensus among participating experts, the element of accessibility received the lowest median scores ranging from 3 to 3.50. Participating experts (100%) agreed that concepts were identifiable in experience and/or practice. While the remaining item for accessibility received a median of 3. All participating experts (100%) agreed that the definitions of the concepts adequately reflected their meanings. These findings are represented in Table 6.

**Table 6.** Accessibility.

Item	Statement	Range	Consensus	Mean	Median	Interquartile range
1	Concepts are identifiable in experience/practice.	3–4	100	3.50	3.50	1
2	Definitions provided for the concepts adequately reflect their meanings.	3–4	100	3.36	3	1

#### Importance

For importance, agreement amongst experts ranged from 85.71% to 100% with six items scoring a median score of 3.50 or higher. All participating experts (100%) agreed that the model presented a general framework to predict the phenomenon of HIV-related stigma and outcomes for child-well-being and that the usage of the developed model would be useful in respective fields and research. While the model was deemed important for the subject area for which it was developed, only 85.71% of the participating experts agreed that it was futuristic and future oriented. These results are indicated in Table 7.

**Table 7.** Importance.

Item	Statement	Range		Mean	Median	Interquartile Range
1	The model has potential to influence current understanding and practice.	3–4	100	3.57	4	1
2	The model may be used to understand the subject area for which it is developed.	3–4	100	3.71	4	1
3	The model provides a general framework in which to act or a means to predict phenomena.	3–4	100	3.64	4	1
4	Given the purpose of the model and its orientation, significant factors have been adequately covered.	3–4	100	3.43	3	1
5	The stated purpose is one that is important to health-related stigma and the well-being of vulnerable children.	3–4	100	3.79	4	1
6	The use of the model will be helpful in respective fields and research.	3–4	100	3.79	4	1
7	The application of the model will resolve issues in research, programmes, and practice.	3–4	100	3.29	3	1
8	The model is futuristic and future looking.	2–4	85.71	3.21	3	1
9	Research based on the model will provide answers to important questions.	3–4	100	3.50	3.50	1

The panel of experts reached consensus for each point of critical reflection as proposed by Chinn and Kramer (2008), thus indicating that the developed model is simplistic, clear, generalisable, accessible, and important. The model is therefore regarded as functional and satisfies the 6-point criteria of critical reflection outlined within the theory generative approach.

## Conclusion

The social transactional model of HIV-related stigma and the psychosocial well-being of COA provide an understanding of the manner in which HIV-related stigma affects the psychosocial well-being of COA. It contributes to the empirical body of knowledge and is the first known model to provide an understanding of HIV-related stigma that is specific to orphaned children and their well-being. It is envisioned that the developed model would guide future research focusing on children in the context of HIV/AIDS. Considering the unique understanding of HIV-related stigma with regards to children, the model may assist with future research studies focusing on the impact of HIV-related stigma on COA and the dissemination of findings. The understanding conveyed by the model would allow for child specific strategies geared towards HIV-related stigma reduction to be appropriately developed and implemented. Furthermore, interventions geared towards equipping COA with healthy coping strategies to lessen the impact of HIV-related stigma on their psychosocial well-being needs to be implemented. As a multidisciplinary approach was used to develop the model, it is accessible and understandable to all disciplines interested in understanding the impact of HIV-related stigma on the psychosocial well-being of COA. The model would enable both policy makers and practitioners to understand COAs experiences of HIV-related stigma and aid the establishment of policies and legislation that addresses the unique needs of COA, affording them sufficient protection against adverse effects and adequate policies addressing their psychosocial needs and challenges. While the developed model makes a noteworthy contribution, it has not been scientifically tested. Therefore, further research assessing the validity of the model is needed.

## Ethical approval

All procedures performed in studies involving human participants were in accordance with the ethical standards of the University of the Western Cape and with the 1964 Helsinki declaration and its later amendments and comparable ethical standards. The study was approved by the University of the Western Cape’s Human and Social Sciences Research Ethics Committee under ethics reference number HS17/1/17.

## Supplementary Material

Conceptual_Framework-_Supp_File_.docx

## Footnotes

1 The information presented in Table 2 is extracted from Yassin (2020).

2 The information presented in Tables 3–7 is extracted from Yassin (2020).
