# Supplementary material for: A model to understand HIV-related stigma and the psychosocial well-being of children orphaned by AIDS: a theory generative approach
Source: SAHARA J. 2021 Oct 15;18(1):131–48. doi: 10.1080/17290376.2021.1989023 (PMC8525949; doi:10.1080/17290376.2021.1989023)
Supplement: Conceptual_Framework-_Supp_File_.docx [file RSAH_A_1989023_SM0081.docx]

**Supplementary File: Conceptual framework material**

The following published works was utilized to develop a conceptual framework from which assumptions about the model were developed.

Adejuwon, G. A., & Oki, S. (2011). Emotional well-being of orphans and vulnerable children in Ogun State orphanages Nigeria: Predictors and implications for policy. *IFE Psychologia: An International Journal*, *19*(1), 1–18.

Alonzo, A. A., & Reynolds, N. R. (1995). Stigma, HIV and AIDS: An exploration and elaboration of a stigma trajectory. *Social Science & Medicine*, *41*(3), 303–315. [doi.org/10.1016/0277-9536(94)00384-6](https://doi.org/10.1016/0277-9536(94)00384-6)

Asikhia, O. A., & Mohangi, K. (2015). A case study of school support and the psychological, emotional and behavioural consequences of HIV and AIDS on adolescents. *SAHARA-J: Journal of Social Aspects of HIV/AIDS*, *12*(1), 123–133. [doi.org/10.1080/17290376.2015.1125305](https://doi.org/10.1080/17290376.2015.1125305)

Audet, C. M., McGowan, C. C., Wallston, K. A., & Kipp, A. M. (2013). Relationship between HIV stigma and self-isolation among people living with HIV in Tennessee. *PloS One*, *8*(8), e69564. [doi.org/10.1371/journal.pone.0069564](https://doi.org/10.1371/journal.pone.0069564)

Bandura, A. (1979). The social learning perspective: Mechanisms of aggression. In H. Toch (Ed.), *Psychology of crime and criminal justice* (pp. 198-236). Prospect Heights, IL, US: Waveland Press.

Bennett, D. S., Traub, K., Mace, L., Juarascio, A., & O'Hayer, C. V. (2016). Shame among people living with HIV: A literature review. *AIDS Care*, *28*(1), 87–91. [doi.org/10.1080/09540121.2015.1066749](https://doi.org/10.1080/09540121.2015.1066749)

Blake, S. M., & Arkin, E. B. (1988). AIDS information monitor: A summary of national public opinion surveys on AIDS: 1983 through 1986. In *AIDS information monitor: A summary of national public opinion surveys on AIDS: 1983 through 1986*. Washington, DC: American Red Cross.

Bogart, L. M., Cowgill, B. O., Kennedy, D., Ryan, G., Murphy, D. A., Elijah, J., & Schuster, M. A. (2008). HIV-related stigma among people with HIV and their families: A qualitative analysis. *AIDS and Behavior*, *12*(2), 244–254. doi:10. 1007/s10461-007-9231-x

Bos, A. E., Pryor, J. B., Reeder, G. D., & Stutterheim, S. E. (2013). Stigma: Advances in theory and research. *Basic and Applied Social Psychology*, *35*(1), 1–9. [doi.org/10.1080/01973533.2012.746147](https://doi.org/10.1080/01973533.2012.746147)

Boyes, M. E., & Cluver, L. D. (2013). Relationships among HIV/AIDS orphanhood, stigma, and symptoms of anxiety and depression in South African youth: A longitudinal investigation using a path analysis framework. *Clinical Psychological Science*, *1*(3), 323–330. doi:10.1177/ 2167702613478595

Boyes, M. E., & Cluver, L. D. (2015). Relationships between familial HIV/AIDS and symptoms of anxiety and depression: The mediating effect of bullying victimization in a prospective sample of South African children and adolescents. *Journal of Youth and Adolescence*, *44*(4), 847–859. doi:10.1007/s10964-014-0146-3

Campbell, C., & Deacon, H. (2006). Unravelling the contexts of stigma: From internalisation to resistance to change. *Journal of Community & Applied Social Psychology*, *16*(6), 411–417. [doi.org/10.1002/casp.901](https://doi.org/10.1002/casp.901)

Campbell, C., & Gibbs, A. (2016). Stigma, gender and HIV: Case studies of inter-sectionality. In J. Boesten & N. K. Poku (Eds.), *Gender and HIV/AIDS: Critical perspectives from the developing world* (pp. 29-46). Farnham, UK: Routledge.

Campbell, C., Skovdal, M., Mupambireyi, Z., & Gregson, S. (2010). Exploring children’s stigmatisation of AIDS-affected children in Zimbabwe through drawings and stories. *Social Science & Medicine*, *71*(5), 975–985. [doi.org/10.1016/j.socscimed.2010.05.028](https://doi.org/10.1016/j.socscimed.2010.05.028)

Campbell, D. T. (1958). Common fate, similarity, and other indices of the status of aggregates of persons as social entities. *Behavioral Science*, *3*(1), 14–25. [doi.org/10.1002/bs.3830030103](https://doi.org/10.1002/bs.3830030103)

Caserta, T. A., Pirttilä-Backman, A. M., & Punamäki, R. L. (2016). Stigma, marginalization and psychosocial well-being of orphans in Rwanda: Exploring the mediation role of social support. *AIDS Care*, *28*(6), 736–744. [doi.org/10.1016/j.bbi.2008.01.007](https://doi.org/10.1016/j.bbi.2008.01.007)

Castro, A., & Farmer, P. (2005). Understanding and addressing AIDS-related stigma: From anthropological theory to clinical practice in Haiti. *American Journal of Public Health*, *95*(1), 53–59. [doi.org/10.2105/AJPH.2003.028563](https://doi.org/10.2105/AJPH.2003.028563)

Chama, S., & Ramirez, O. (2015). Psychosocial challenges of young people affected by HIV: Experiences from Hamilton County, Chattanooga, Tennessee. *AIDS Care*, *27*(6), 789–795. doi.org/10.1080/09540121.2014.989807.

Chaudoir, S. R., Earnshaw, V. A., & Andel, S. (2013). “Discredited” versus “discreditable”: Understanding how shared and unique stigma mechanisms affect psychological and physical health disparities. *Basic and Applied Social Psychology*, *35*(1), 75–87. [doi.org/10.1080/01973533.2012.746612](https://doi.org/10.1080/01973533.2012.746612)

Cheney, K. E. (2015). Suffering, silence, and status: The importance and challenges of qualitative research on AIDS orphanhood. *AIDS Care*, *27*(1), 38–40. [doi.org/10.1080/09540121.2014.963010](https://doi.org/10.1080/09540121.2014.963010)

Chi, P., Li, X., Du, H., Tam, C. C., Zhao, J., & Zhao, G. (2016). Does stigmatization wear down resilience? A longitudinal study among children affected by parental HIV. *Personality and Individual Differences*, *96*, 159–163. [doi.org/10.1016/j.paid.2016.03.001](https://doi.org/10.1016/j.paid.2016.03.001)

Chi, P., Slatcher, R. B., Li, X., Zhao, J., Zhao, G., Ren, X., ... & Stanton, B. (2015). Perceived stigmatization, resilience, and diurnal cortisol rhythm among children of parents living with HIV. *Psychological Science*, *26*(6), 843–852. [doi.org/10.1177%2F0956797615572904](https://doi.org/10.1177%252F0956797615572904)

Cluver, L. D., Gardner, F., & Operario, D. (2008). Effects of stigma on the mental health of adolescents orphaned by AIDS. *Journal of Adolescent Health*, *42*(4), 410–417. [doi.org/10.1016/j.jadohealth.2007.09.022](https://doi.org/10.1016/j.jadohealth.2007.09.022)

Cree, V. E., Kay, H., Tisdall, K., & Wallace, J. (2004). Stigma and parental HIV. *Qualitative Social Work: Research and Practice*, *3*(1), 7–24. [doi.org/10.1177%2F1473325004041129](https://doi.org/10.1177%252F1473325004041129)

Crocker, J., Major, B., Steele, C. (1998). Social Psychology. In S. Fiske, D. Gilbert, & G. Lindzy. (Eds). *The handbook of social psychology* (pp. 504-53). Boston: McGraw-Hill.

Dahl, B. (2009). The “failures of culture”: Christianity, kinship, and moral discourses about orphans during Botswana's AIDS crisis. *Africa Today*, *56*(1), 22–43. doi: 10.2979/aft.2009.56.1.22.

Dahlui, M., Azahar, N., Bulgiba, A., Zaki, R., Oche, O. M., Adekunjo, F. O., & Chinna, K. (2015). HIV/AIDS related stigma and discrimination against PLWHA in Nigerian population. *PloS One*, *10*(12), e0143749. [doi.org/10.1371/journal.pone.0143749](https://doi.org/10.1371/journal.pone.0143749)

Deacon, H. (2006). Towards a sustainable theory of health‐related stigma: Lessons from the HIV/AIDS literature. *Journal of community & applied social psychology*, *16*(6), 418–425. [doi.org/10.1002/casp.900](https://doi.org/10.1002/casp.900)

Deacon, H., & Stephney, I. (2007). *HIV/AIDS, stigma and children: A literature review*. Cape Town: HSRC Press.

de Witt, M. W., & Lessing, A. C. (2010). The psychosocial well-being of orphans in Southern Africa: The perception of orphans and teachers. *TD: The Journal for Transdisciplinary Research in Southern Africa*, *6*(2), 461–477. doi 10.4102/td.v6i2.262

DeNeve, K. M., & Harris, C. (1998). The happy personality: a meta-analysis of 137 personality traits and subjective well-being. *Psychological Bulletin*, *124*(2), 197–229.

Doku, P. N., & Minnis, H. (2016). Multi-informant perspective on psychological distress among Ghanaian orphans and vulnerable children within the context of HIV/AIDS. *Psychological Medicine*, *46*(11), 2329–2336. [doi.org/10.1017/S0033291716000829](https://doi.org/10.1017/S0033291716000829)

Earnshaw, V. A., & Kalichman, S. C. (2013). Stigma experienced by people living with HIV/AIDS. In P. Liamputtong (Ed.), *Stigma, discrimination and living with HIV/AIDS* (pp. 23–38). Dordrecht: Springer.

Earnshaw, V. A., Smith, L. R., Chaudoir S. R., Amico, K. R., Copenhaver M. M. (2013). HIV stigma mechanisms and well-being among PLWH: A test of the HIV stigma framework. *AIDS and Behavior*, *17*(5), 1785–1795. [doi.org/10.1007%2Fs10461-013-0437-9](https://dx.doi.org/10.1007%252Fs10461-013-0437-9)

Earnshaw, V. A., Smith, L. R., Shuper, P. A., Fisher, W. A., Cornman, D. H., & Fisher, J. D. (2014). HIV stigma and unprotected sex among PLWH in KwaZulu-Natal, South Africa: A longitudinal exploration of mediating mechanisms. *AIDS Care*, *26*(12), 1506–1513. [doi.org/10.1080/09540121.2014.938015](https://doi.org/10.1080/09540121.2014.938015)

Elafros, M. A., Gardiner, J. C., Sikazwe, I., Okulicz, J. F., Paneth, N., Chomba, E., & Birbeck, G. L. (2018). Evaluating layered stigma from comorbid HIV and epilepsy among Zambian adults. *eNeurologicalSci*, *13*, 56–62. [doi.org/10.1016/j.ensci.2017.12.001](https://doi.org/10.1016/j.ensci.2017.12.001)

Florom‐Smith, A. L., & De Santis, J. P. (2012). Exploring the concept of HIV‐related stigma. *Nursing Forum*, *47*(3), 153–165. [doi.org/10.1111/j.1744-6198.2011.00235.x](https://doi.org/10.1111/j.1744-6198.2011.00235.x)

Fuller-Rowell, T. E., Doan, S. N., & Eccles, J. S. (2012). Differential effects of perceived discrimination on the diurnal cortisol rhythm of African Americans and Whites. *Psychoneuroendocrinology*, *37*(1), 107–118. [doi.org/10.1016/j.psyneuen.2011.05.011](https://doi.org/10.1016/j.psyneuen.2011.05.011)

Gabe, J., Bury, M., & Elston, M. A. (2004). *Key concepts in medical sociology*. London: Sage.

Galvan, F. H., Davis, E. M., Banks, D., & Bing, E. G. (2008). HIV stigma and social support among African Americans. *AIDS Patient Care and STDs*, *22*(5), 423–436. [doi.org/10.1089/apc.2007.0169](https://doi.org/10.1089/apc.2007.0169)

Gamarel, K. E., Kuo, C. C., Boyes, M. E., & Cluver, L. D. (2017). The dyadic effects of HIV stigma on the mental health of children and their parents in South Africa. *Journal of HIV/AIDS & Social Services*, *16*(4), 351–366. [doi.org/10.1080/15381501.2017.1320619](https://doi.org/10.1080/15381501.2017.1320619)

Genberg, B. L., Hlavka, Z., Konda, K. A., Maman, S., Chariyalertsak, S., Chingono, A., ... & Celentano, D. D. (2009). A comparison of HIV/AIDS-related stigma in four countries: Negative attitudes and perceived acts of discrimination towards people living with HIV/AIDS. *Social Science & Medicine*, *68*(12), 2279–2287. [doi.org/10.1016/j.socscimed.2009.04.005](https://doi.org/10.1016/j.socscimed.2009.04.005)

Gilbert, L., & Walker, L. (2010). ‘My biggest fear was that people would reject me once they knew my status…’: stigma as experienced by patients in an HIV/AIDS clinic in Johannesburg, South Africa. *Health & Social Care in the Community*, *18*(2), 139–146. [doi.org/10.1111/j.1365-2524.2009.00881.x](https://doi.org/10.1111/j.1365-2524.2009.00881.x)

Goffman, E. (1963). *Stigma: Notes on the management of spoiled identity*. New York: Simon & Shuster.

Hatzenbuehler, M. L. (2016). Structural stigma: Research evidence and implications for psychological science. *American Psychologist*, *71*(8), 742–751. doi [10.1037/amp0000068](https://doi.org/10.1037/amp0000068)

Herek, G. M. (1999). AIDS and stigma. *American Behavioral Scientist, 42*(7), 1106–1116. [doi.org/10.1177%2F0002764299042007004](https://doi.org/10.1177%252F0002764299042007004)

Herek, G. M., Capitanio, J. P., & Widaman, K. F. (2002). HIV-related stigma and knowledge in the United States: Prevalence and trends, 1991–1999. *American Journal of Public Health*, *92*(3), 371–377. [doi.org/10.2105/AJPH.92.3.371](https://doi.org/10.2105/AJPH.92.3.371)

Ishikawa, N., Pridmore, P., Carr-Hill, R., & Chaimuangdee, K. (2011). The attitudes of primary schoolchildren in Northern Thailand towards their peers who are affected by HIV and AIDS. *AIDS Care*, *23*(2), 237–244. [doi.org/10.1080/09540121.2010.507737](https://doi.org/10.1080/09540121.2010.507737)

Jones, E. E., Farina, A., Hastorf, A. H., Markus, H. M., Miller, D. T., & Scott, R. A. (1984). *Social stigma: The psychology of marked relationships*. New York: Freeman.

Kalichman, S., Katner, H., Banas, E., & Kalichman, M. (2017). Population density and AIDS-related stigma in large-urban, small-urban, and rural communities of the southeastern USA. *Prevention Science*, *18*(5), 517–525. [doi.org/10.1007%2Fs11121-017-0761-9](https://dx.doi.org/10.1007%252Fs11121-017-0761-9)

Katz, I. (1979). Some thoughts about the stigma notion. *Personality and Social Psychology Bulletin*, *5*(4), 447–460. [doi.org/10.1177%2F014616727900500406](https://doi.org/10.1177%252F014616727900500406)

Kheswa, G., & Duncan, N. (2011). The stigmatisation of children affected by HIV and AIDS in a Gauteng township area. *Child Abuse Research in South Africa*, *12*(1), 38–50. <https://hdl.handle.net/10520/EJC24199>

Kimane, I. (2005). Update on the situation analysis of orphaned and vulnerable children in Lesotho. Unpublished Report submitted to the Ministry of Health and Social Welfare. Lesotho and Save the Children and UNICEF.

Kittikorn, N., Street, A. F., & Blackford, J. (2006). Managing shame and stigma: Case studies of female carers of people with AIDS in Southern Thailand. *Qualitative Health Research*, *16*(9), 1286–1301. [doi.org/10.1177%2F1049732306293992](https://doi.org/10.1177%252F1049732306293992)

Klonoff, E. A. (2014). Introduction to the special section on discrimination. *Health Psychology*, *33*(1), 1-2. [doi/10.1037/hea0000070](https://psycnet.apa.org/doi/10.1037/hea0000070)

Lalthapersad-Pillay, P. (2008). The orphan problem in selected African countries. *Africa Insight*, *37*(4), 148–159.

Lata, S., & Verma, S. (2013). Mental health of HIV/AIDS orphans: A review. *Journal of AIDS and HIV Research*, *5*(12), 455–467. doi: 10.5897/JAHR2013.0271

Li, X., Chi, P., Sherr, L., Cluver, L., & Stanton, B. (2015). Psychological resilience among children affected by parental HIV/AIDS: A conceptual framework. *Health Psychology and Behavioral Medicine*, *3*(1), 217–235. [doi.org/10.1080/21642850.2015.1068698](https://doi.org/10.1080/21642850.2015.1068698)

Li, X., Harrison, S. E., Fairchild, A. J., Chi, P., Zhao, J., & Zhao, G. (2017). A randomized controlled trial of a resilience-based intervention on psychosocial well-being of children affected by HIV/AIDS: Effects at 6-and 12-month follow-up. *Social Science & Medicine*, *190*, 256–264. [doi.org/10.1016/j.socscimed.2017.02.007](https://doi.org/10.1016/j.socscimed.2017.02.007)

Li, X., Naar-King, S., Barnett, D., Stanton, B., Fang, X., & Thurston, C. (2008). A developmental psychopathology framework of the psychosocial needs of children orphaned by HIV. *Journal of the Association of Nurses in AIDS Care*, *19*(2), 147–157. [doi.org/10.1016/j.jana.2007.08.004](https://doi.org/10.1016/j.jana.2007.08.004)

Lin, X., Zhao, G., Li, X., Stanton, B., Zhang, L., Hong, Y., … Fang, X. (2010). Perceived HIV stigma among children in a high HIV-prevalence area in central China: Beyond the parental HIV-related illness and death. *AIDS Care*, *22*(5), 545–555. doi: 10.1080/09540120903253999.

Link, B. G., & Phelan, J. C. (2001). Conceptualizing stigma. *Annual Review of Sociology*, *27*(1), 363–385. [doi.org/10.1146/annurev.soc.27.1.363](https://doi.org/10.1146/annurev.soc.27.1.363)

Louw, D., Mokhosi, M., & Van den Berg, H. (2012). Stressors, social resources and coping skills among double aids-orphaned adolescents. *Child Abuse Research in South Africa*, *13*(2), 1–12. <https://hdl.handle.net/10520/EJC128385>

Lyimo, R. A., Stutterheim, S. E., Hospers, H. J., de Glee, T., van der Ven, A., & de Bruin, M. (2014). Stigma, disclosure, coping, and medication adherence among people living with HIV/AIDS in Northern Tanzania. *AIDS Patient Care and STDs*, *28*(2), 98–105. [doi.org/10.1089/apc.2013.0306](https://doi.org/10.1089/apc.2013.0306)

Major, B., & O'Brien, L. T. (2005). The social psychology of stigma. *Annual Review of Psychology*, *56*, 393–421. [doi.org/10.1146/annurev.psych.56.091103.070137](https://doi.org/10.1146/annurev.psych.56.091103.070137)

Makoae, L. N., Greeff, M., Phetlhu, R. D., Uys, L. R., Naidoo, J. R., Kohi, T. W., ... & Holzemer, W. L. (2008). Coping with HIV-related stigma in five African countries. *Journal of the Association of Nurses in AIDS Care*, *19*(2), 137–146. [doi.org/10.1016/j.jana.2007.11.004](https://doi.org/10.1016/j.jana.2007.11.004)

Mason, S., & Sultzman, V. O. (2019). Stigma as experienced by children of HIV-positive parents: A narrative review. *AIDS Care*, *31*(9), 1049–1060. [doi.org/10.1080/09540121.2019.1573968](https://doi.org/10.1080/09540121.2019.1573968)

Mason, S., Sultzman, V., & Berger, B. (2014). “Like being in a cage”: stigma as experienced by adolescents whose mothers are living with HIV. *Vulnerable Children and Youth Studies*, *9*(4), 323–331. doi:10.1080/17450128.2014.933941

Mawar, N., Sahay, S., Pandit, A., & Mahajan, U. (2005). The third phase of HIV pandemic: social consequences of HIV/AIDS stigma & discrimination & future needs. *Indian Journal of Medical Research*, *122*(6), 471–484.

McAteer, C. I., Truong, N. A. T., Aluoch, J., Deathe, A. R., Nyandiko, W. M., Marete, I., & Vreeman, R. C. (2016). A systematic review of measures of HIV/AIDS stigma in paediatric HIV‐infected and HIV‐affected populations. *Journal of the International AIDS Society*, *19*(1), 21204–21211. [doi.org/10.7448%2FIAS.19.1.21204](https://dx.doi.org/10.7448%252FIAS.19.1.21204)

McHenry, M. S., Nyandiko, W. M., Scanlon, M. L., Fischer, L. J., McAteer, C. I., Aluoch, J., Naanyu, V., & Vreeman, R. C. (2017). HIV stigma: Perspectives from Kenyan child caregivers and adolescents living with HIV. *Journal of the International Association of Providers of AIDS Care (JIAPAC)*, *16*(3), 215–225. [doi.org/10.1177%2F2325957416668995](https://doi.org/10.1177%252F2325957416668995)

Messer, L. C., Pence, B. W., Whetten, K., Whetten, R., Thielman, N., O'Donnell, K., & Ostermann, J. (2010). Prevalence and predictors of HIV-related stigma among institutional-and community-based caregivers of orphans and vulnerable children living in five less-wealthy countries. *BMC Public Health*, *10*(1), 504–511. [doi.org/10.1186/1471-2458-10-504](https://doi.org/10.1186/1471-2458-10-504)

Mo, P. K., Lau, J. T., Yu, X., & Gu, J. (2015). A model of associative stigma on depression and anxiety among children of HIV-infected parents in China. *AIDS and Behavior*, *19*(1), 50–59. [doi.org/10.1007/s10461-014-0809-9](https://doi.org/10.1007/s10461-014-0809-9)

Ntozi, J. P., Ahimbisibwe, F. E., Odwee, J. O., Ayiga, N., & Okurut, F. N. (1999). Orphan care: The role of the extended family in northern Uganda. In I. O. Orubuloye, J. Cakdwell, & J. P. Ntozi (Eds.), *The continuing HIV/AIDS epidemic in Africa: Responses and coping strategies* (pp. 225–236). Canberra: Health Transition Centre.

Nyamukapa, C. A., Gregson, S., Lopman, B., Saito, S., Watts, H. J., Monasch, R., & Jukes, M. C. (2008). HIV-associated orphanhood and children’s psychosocial distress: Theoretical framework tested with data from Zimbabwe. *American Journal of Public Health*, *98*(1), 133–141. [doi.org/10.2105/AJPH.2007.116038](https://doi.org/10.2105/AJPH.2007.116038)

Ogden, J., & Nyblade, L. (2005). Common at its core: HIV-related stigma across contexts. Washington, DC: International Center for Research on Women.

Ogina, T. A. (2012). The use of drawings to facilitate interviews with orphaned children in Mpumalanga province, South Africa. *South African Journal of Education*, *32*(4), 428–440. [doi.org/10.15700/saje.v32n4a668](http://dx.doi.org/10.15700/saje.v32n4a668)

Ornacka, K. (2014). Children of “Vulnerable Identity” – Do They Have To Be Excluded? Some Reflections Based on Empirical Research. *Acta Universitatis Lodziensis. Folia Sociologica, 49,* 81–95. http://hdl.handle.net/11089/7343

Parker, R., & Aggleton, P. (2003). HIV and AIDS-related stigma and discrimination: A conceptual framework and implications for action. *Social Science & Medicine*, *57*(1), 13–24. [doi: 10.1016/S0277-9536(02)00304-0](https://doi.org/10.1016/S0277-9536(02)00304-0)

Pouw, N., & Hodgkinson, K. (2016). SOS Children’s Villages Literature Review: The Social Exclusion of Vulnerable Youth. Amsterdam Institute for Social Science Research. Amsterdam: University of Amsterdam.

Prati, G., & Pietrantoni, L. (2009). Optimism, social support, and coping strategies as factors contributing to posttraumatic growth: A meta-analysis. *Journal of Loss and Trauma*, *14*(5), 364–388. [doi.org/10.1080/15325020902724271](https://doi.org/10.1080/15325020902724271)

Pryor, J. B., Reeder, G. D., & Monroe, A. E. (2012). The infection of bad company: Stigma by association. *Journal of Personality and Social Psychology*, *102*(2), 224–241. [doi: 10.1037/a0026270](https://psycnet.apa.org/doi/10.1037/a0026270)

Rao, D., Feldman, B. J., Fredericksen, R. J., Crane, P. K., Simoni, J. M., Kitahata, M. M., & Crane, H. M. (2012). A structural equation model of HIV-related stigma, depressive symptoms, and medication adherence. *AIDS and Behavior*, *16*(3), 711–716. [doi: 10.1007/s10461-011-9915-0](https://doi.org/10.1007/s10461-011-9915-0)

Rushing, W. A. (2018). *The AIDS epidemic: Social dimensions of an infectious disease*. New York: Routledge.

Scambler G. (1989). *Epilepsy*. London: Routledge.

Scambler, G., & Paoli, F. (2008). Health work, female sex workers and HIV/AIDS: Global and local dimensions of stigma and deviance as barriers to effective interventions. *Social Science & Medicine*, *66*(8), 1848–1862. [doi.org/10.1016/j.socscimed.2008.01.002](https://doi.org/10.1016/j.socscimed.2008.01.002)

Schibalski, J. V., Müller, M., Ajdacic-Gross, V., Vetter, S., Rodgers, S., Oexle, N., ... & Ruesch, N. (2017). Stigma-related stress, shame and avoidant coping reactions among members of the general population with elevated symptom levels. *Comprehensive Psychiatry*, *74*, 224–230. [doi.org/10.1016/j.comppsych.2017.02.001](https://doi.org/10.1016/j.comppsych.2017.02.001)

Sengupta, S., Strauss, R. P., Miles, M. S., Roman-Isler, M., Banks, B., & Corbie-Smith, G. (2010). A conceptual model exploring the relationship between HIV stigma and implementing HIV clinical trials in rural communities of North Carolina. *North Carolina Medical Journal*, *71*(2), 113–122.

Stangl, A. L., Lloyd, J. K., Brady, L. M., Holland, C. E., & Baral, S. (2013). A systematic review of interventions to reduce HIV‐related stigma and discrimination from 2002 to 2013: How far have we come? *Journal of the International AIDS Society*, *16*(3 Suppl 2*)*, 18734. [doi.org/10.7448/IAS.16.3.18734](https://doi.org/10.7448/IAS.16.3.18734)

Steward, W. T., Herek, G. M., Ramakrishna, J., Bharat, S., Chandy, S., Wrubel, J., & Ekstrand, M. L. (2008). HIV-related stigma: adapting a theoretical framework for use in India. *Social Science & Medicine*, *67*(8), 1225–1235. [doi.org/10.1016/j.socscimed.2008.05.032](https://doi.org/10.1016/j.socscimed.2008.05.032)

Stuenkel, D, & Wong, V. (2009). Stigma. In D. Stuenkel, V. Wong, P. Larsen, & I. Lubkin (Eds.), *Chronic illness: Impact and Intervention* (pp. 47-74). Boston: Jones & Bartlett Learning.

Stutterheim, S. E., Bos, A. E., Shiripinda, I., de Bruin, M., Pryor, J. B., & Schaalma, H. P. (2012). HIV-related stigma in African and Afro-Caribbean communities in the Netherlands: Manifestations, consequences and coping. *Psychology & Health*, *27*(4), 395–411. [doi.org/10.1080/08870446.2011.585426](https://doi.org/10.1080/08870446.2011.585426)

Subbarao, K., & Coury, D. (2004). *Reaching out to Africa's orphans: A framework for public action*. Washington, DC: The World Bank.

Tanga, P. T. (2013). The impact of the declining extended family support system on the education of orphans in Lesotho. *African Journal of AIDS Research*, *12*(3), 173–183. [doi.org/10.2989/16085906.2013.863217](https://doi.org/10.2989/16085906.2013.863217)

Thornton, A. (2001). Introduction and overview. In A. Thornton (Ed.), *The well-being of children and families: Research and data needs* (pp. 3–27)*.* Ann Arbor, MI: University of Michigan Press.

Thupayagale-Tshweneagae, G., & Benedict, S. (2011). The burden of secrecy among South African adolescents orphaned by HIV and AIDS. *Issues in Mental Health Nursing*, *32*(6), 355–358. [doi.org/10.3109/01612840.2011.576128](https://doi.org/10.3109/01612840.2011.576128)

Tran, T. T. N., & Mwanri, L. (2013). Addressing stigma and discrimination in HIV/AIDS affected orphans and vulnerable children in Vietnam. *World Journal of Preventive Medicine*, *1*(3), 30–35. doi:10.12691/jpm-1-3-4.

Treves-Kagan, S., Steward, W. T., Ntswane, L., Haller, R., Gilvydis, J. M., Gulati, H., ... & Lippman, S. A. (2015). Why increasing availability of ART is not enough: a rapid, community-based study on how HIV-related stigma impacts engagement to care in rural South Africa. *BMC Public Health*, *16*(87), 1-13. doi.org/10.1186/s12889-016-2753-2.

Tsai, A. C., Bangsberg, D. R., Kegeles, S. M., Katz, I. T., Haberer, J. E., Muzoora, C., ... & Weiser, S. D. (2013). Internalized stigma, social distance, and disclosure of HIV seropositivity in rural Uganda. *Annals of Behavioral Medicine*, *46*(3), 285–294. [doi.org/10.1007/s12160-013-9514-6](https://doi.org/10.1007/s12160-013-9514-6)

Turan, J. M., Elafros, M. A., Logie, C. H., Banik, S., Turan, B., Crockett, K. B., Pescosolido, B., & Murray, S. M. (2019). Challenges and opportunities in examining and addressing intersectional stigma and health. *BMC Medicine*, *17*(7), 1-15. [doi.org/10.1186/s12916-018-1246-9](https://doi.org/10.1186/s12916-018-1246-9)

UNAIDS. (2000). *AIDS and HIV Infection: Information for United Nations Employees and their Families*. Geneva, Switzerland.

Williams, L. D., & Aber, J. L. (2019). The Multilevel Relationships of HIV‐Related Stigma to Child and Caregiver Mental Health among HIV‐Affected Households in South Africa. *American Journal of Community Psychology*, *63*(1-2), 3–16. [doi.org/10.1002/ajcp.12280](https://doi.org/10.1002/ajcp.12280)

Xanthopoulos, M. S., & Daniel, L. C. (2013). Coping and social support. In A. M. Nezu, C. M. Nezu, P. A. Geller, I. B. Weiner, A. M. Nezu, C. M. Nezu, … I. B. Weiner (Eds.), *Handbook of psychology, Vol. 9: Health Psychology* (2nd ed. pp. 57–78). Hoboken, NJ: Wiley.

Zhao, J., Li, X., Fang, X., Hong, Y., Zhao, G., Lin, X., Zhang, L., & Stanton, B. (2010). Stigma against children affected by AIDS (SACAA): Psychometric evaluation of a brief measurement scale. *AIDS and Behavior*, *14*(6), 1302–1312. [doi.org/10.1007/s10461-009-9629-8](https://doi.org/10.1007/s10461-009-9629-8)

Zhao, G., Li, X., Fang, X., Zhao, J., Yang, H., & Stanton, B. (2007). Care arrangements, grief and psychological problems among children orphaned by AIDS in China. *AIDS Care*, *19*(9), 1075–1082. [doi.org/10.1080/09540120701335220](https://doi.org/10.1080/09540120701335220)
